# Factors associated with insomnia in hemodialysis patients: A protocol for systematic review and meta-analysis

**DOI:** 10.1371/journal.pone.0343236

**Published:** 2026-02-20

**Authors:** Shenao Yu, Xinling Zhou, Liuyan Xu, Lingli Fang, Yanru Wang

**Affiliations:** School of Nursing, Zhejiang Chinese Medical University, Hangzhou, Zhejiang, China; Japanese Red Cross Medical Center, JAPAN

## Abstract

**Introduction:**

Insomnia is a prevalent sleep disorder among hemodialysis (HD) patients, significantly impairing quality of life and increasing mortality risk. However, current evidence regarding the factors associated with insomnia in this population remains inconsistent. This systematic review and meta-analysis aim to comprehensively identify and synthesize the physiological, psychological, and dialysis-related factors associated with insomnia in HD patients.

**Methods:**

We will systematically search PubMed, Web of Science, Cochrane Library, EMBASE, CINAHL, CNKI, SinoMed, and Wanfang Data from inception to February 1, 2026. We will include observational studies (cohort, case-control, and cross-sectional) investigating potential associated factors for insomnia in adult HD patients. Two reviewers will independently screen titles, abstracts, and full texts, extract data, and assess the risk of bias using the Newcastle-Ottawa Scale and the Agency for Healthcare Research and Quality methodology. Data synthesis will be performed using RevMan 5.4 and Stata 16.0 software. We will calculate Odds Ratios and Mean Differences with 95% Confidence Intervals. Given the anticipated heterogeneity, a random-effects model will be employed. Subgroup and sensitivity analyses will be conducted to explore sources of heterogeneity. The certainty of the evidence will be evaluated using the GRADE system.

**Discussion:**

This study will represent a comprehensive systematic review integrating factors associated with insomnia in HD patients. By applying the GRADE system, this review will systematically evaluate the strength and certainty of evidence for each identified factor. The anticipated findings will guide the development of targeted preventive strategies and personalized management protocols, ultimately improving sleep quality and prognosis for HD patients.

**Trial registration:**

PROSPERO (CRD420251241385).

## Introduction

Currently, over 3 million patients with kidney failure undergo maintenance dialysis globally [1], with hemodialysis (HD) accounting for approximately 91% of these cases as the predominant renal replacement therapy [2]. While HD effectively sustains life by removing metabolic waste and maintaining electrolyte balance [3], the focus of clinical management has traditionally prioritized acute complications, such as electrolyte disturbances and heart failure. Consequently, chronic distressing symptoms, including insomnia, pruritus, and constipation, are frequently overshadowed and under-addressed in clinical practice [4].

Among these chronic complications, insomnia is the most prevalent sleep complaint in HD patients, characterized by difficulty initiating sleep, maintaining sleep, or early awakening [5]. Recent data suggest that up to 46% of HD patients suffer from insomnia [6]. This condition is not merely a quality-of-life issue; it is independently associated with cognitive decline, psychiatric disorders (depression and anxiety) [7,8], increased cardiovascular risk, and elevated all-cause mortality [9–12], thereby imposing a substantial economic burden on healthcare systems and society [13].

Despite the high prevalence of insomnia, the factors associated with insomnia in this population remain unclear and controversial. Existing literature suggests a multidimensional etiology involving: physiological factors, such as pruritus caused by uremic toxin accumulation, restless legs syndrome (RLS) [14], and chronic pain; psychological factors, including the burden of disease and emotional disorders [15]; and dialysis-related factors. Although HD partially clears toxins, it disrupts patients’ lifestyle and circadian rhythms. Studies indicate that HD patients experience significantly shortened total sleep time and reduced sleep efficiency, with marked sleep fragmentation [1,16]. A potential contributing mechanism is intradialytic napping, which affects nearly half of patients and disrupts homeostatic sleep drive [17]. Furthermore, dialysis vintage [18], dialysis modality [19], and dietary habits [20] are potential influencers. However, findings across studies regarding these factors are inconsistent and lack systematic quantitative synthesis.

Although systematic reviews and meta-analyses regarding sleep quality in patients with kidney disease already exist, they have primarily focused on the prevalence of sleep disorders or conflated different renal replacement therapies (hemodialysis, peritoneal dialysis, and kidney transplantation) [6,21]. Few studies have systematically synthesized specific associated factors within the hemodialysis population. Furthermore, a substantial body of evidence from Chinese databases remains largely inaccessible to international readers. Therefore, this review aims to bridge these gaps; identifying factors associated with insomnia in hemodialysis patients is crucial for reducing its incidence, improving prognosis, and optimizing clinical management.

## Methods

### Study registration

The protocol for this systematic review has been registered in the PROSPERO International Prospective Register of Systematic Reviews (Registration number: CRD420251241385). We will strictly adhere to the Preferred Reporting Items for Systematic Review and Meta-Analysis Protocols (PRISMA-P) guidelines (S1 File) [22] and the Meta-analysis of Observational Studies in Epidemiology (MOOSE) guidelines [23]. The final report will be drafted in accordance with the PRISMA statement [24].

### Ethics and dissemination

As this study is a secondary analysis based on published literature, neither patient informed consent nor ethical committee approval is required. The findings of this systematic review and meta-analysis will be disseminated through publication in a peer-reviewed journal.

### Inclusion criteria

#### Participants.

We will include adult patients (aged ≥ 18 years) who have undergone HD for at least three months. To ensure valid associated factor analysis, studies must involve HD patients and allow for comparisons between those with insomnia and those without insomnia. Insomnia must be identified using validated self-reported scales with established cut-off values, specifically: the Pittsburgh Sleep Quality Index (PSQI) score ≥5 [25], the Athens Insomnia Scale (AIS) score ≥6 [26], or the Insomnia Severity Index (ISI) score >13 [27]. Studies employing clinical diagnoses based on standard criteria (DSM-5, ICSD-3) will also be included [5].

#### Exposure.

The primary outcome measures of this study are baseline characteristics potentially associated with the onset or exacerbation of insomnia. To ensure the interpretability of pooled results, we categorized potential associated factors into four distinct domains, including but not limited to (1) Sociodemographic characteristics, such as age, sex, race, and body mass index (BMI); (2) Dialysis-related characteristics, including dialysis vintage, medication history, primary disease, dialysis shift (morning, afternoon, and evening groups) [18], and dialysis modality (high-flux hemodialysis, low-flux hemodialysis, and hemodiafiltration) [28]; (3) Comorbidities, such as pruritus, RLS, depression; (4) other health factors, such as dietary habits and lifestyle. For the purpose of this review, dialysis modality refers to the classification based on membrane flux and solute transport mechanisms, specifically categorized into low-flux hemodialysis, high-flux hemodialysis, and hemodiafiltration.

#### Study types.

This study will strictly include observational studies, specifically case-control studies, cohort studies, and cross-sectional studies.

### Exclusion criteria

Studies meeting any of the following criteria will be excluded: (1) Full text is unavailable and cannot be obtained despite contacting the authors; (2) Duplicate publications, conference abstracts, meta-analyses, reviews, protocols, animal studies, and editorials.

### Search strategy

We will comprehensively search the following databases: PubMed, Web of Science, Cochrane Library, CINAHL, EMBASE, CNKI, SinoMed, and Wanfang Data. No language restrictions will be applied to the search settings. The search timeframe is from database inception to February 1, 2026. The search strategy will utilize a combination of Medical Subject Headings (MeSH) and free-text terms, connected with Boolean operators (AND, OR), and adapted to the syntax of each database. Additionally, we will search grey literature via Google Scholar (https://scholar.google.com) and manually screen the reference lists of included articles to avoid omissions. We will screen the top 200 records from the Google Scholar search results by relevance [29]. The detailed search strategy is provided in Supplementary S2 File. The search terms primarily encompass “hemodialysis,” “insomnia,” and “associated factors” or “risk factors.”

### Data collection and analysis

**Fig 1:**
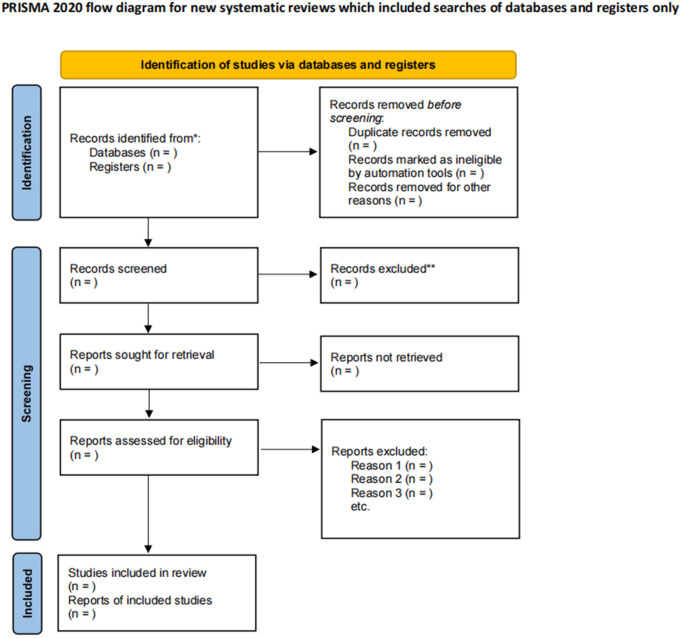
Flowchart of studies included in the systematic review.

All retrieved records will be imported into EndNote 21 software. Two trained reviewers (LY and SA) will independently screen the literature. First, duplicates and clearly irrelevant studies will be removed by screening titles and abstracts. Subsequently, the full texts of the remaining articles will be reviewed to determine final eligibility. For articles in languages other than English or Chinese, we will use translation software (Google Translate https://translate.google.com) for initial screening. If a study is deemed relevant, the full text will be translated to assess eligibility. While we acknowledge the potential for minor inaccuracies with automated translation, this method will be strictly limited to initial screening. Subsequent data extraction from other full texts will be conducted or verified by fluent speakers of the respective language to ensure accuracy. Any discrepancies between the two reviewers will be resolved through discussion or consultation with a third reviewer (YR). The study selection process is illustrated in Fig 1.

### Data extraction

We will design a data extraction form using Microsoft Excel to collect the following information: (1) Study characteristics: First author, publication year, study design, and country/region. (2) Patient and outcome data: Sample size, sex, age, primary disease, dialysis vintage, dialysis shift, dialysis modality, medication use, comorbidities, and the effect sizes of associated factors (Odds Ratios [ORs] or Risk Ratios [RRs]) with their 95% confidence intervals (CIs). Data extraction will be performed independently by two reviewers (SA and LY), with discrepancies resolved by a third reviewer (YR). In cases of missing data, we will attempt to contact the corresponding authors. If data remain unobtainable, we will perform sensitivity analyses by excluding studies with missing data to assess the robustness of the pooled results, and we will discuss the potential impact of missing data in the narrative synthesis.

### Risk of bias assessment

Two qualified reviewers (SA and XL) will independently assess the quality of included studies. The quality of cohort and case-control studies will be evaluated using the Newcastle-Ottawa Scale (NOS) [30]. The NOS comprises three domains: selection, comparability, and outcome (for cohort studies) or exposure (for case-control studies). It consists of eight items; a maximum of one point is awarded for each item, except for the comparability domain, which allows for a maximum of two points. The total score ranges from 0 to 9, with scores interpreted as follows: 0–4 indicates low quality, 5–6 indicates moderate quality, and ≥7 indicates high quality. The quality of cross-sectional studies will be assessed using the criteria recommended by the Agency for Healthcare Research and Quality (AHRQ) [31].This scale comprises 11 items; each item is scored 1 point for a response of ‘Yes’ and 0 points for ‘No’ or ‘Unclear’. The total score ranges from 0 to 11, with quality classified as low (0–3), moderate (4–7), or high (8–11). Studies classified as low quality (NOS < 5 or AHRQ < 4) will be retained in the descriptive analysis but will be examined in sensitivity analyses to assess their impact on the overall effect size. Any discrepancies in scoring will be adjudicated by a third reviewer (YR).

### Strategy for data synthesis

Meta-analysis will be performed using RevMan 5.4 and Stata 16.0 software. Quantitative synthesis will be performed only when at least three studies report the same exposure-outcome association using sufficiently similar definitions and cut-off values. Continuous variables will be expressed as the Standardized Mean Differences (SMDs) or Mean Differences (MDs) with 95% CIs. Categorical variables will be expressed as ORs or RRs with 95% CIs, with the level of statistical significance set at P < 0.05. We will prioritize the analysis of adjusted ORs or RRs derived from multivariable analyses to control for confounding factors. A qualitative narrative synthesis will be conducted when fewer than three studies address a specific factor, when significant discrepancies exist in exposure definitions or outcome measures, or when only unadjusted univariate data are available.

### Heterogeneity assessment

We will assess heterogeneity using the Higgins I^2^ statistic and Cochran’s Q test [32]. The I^2^ statistic will be used to evaluate the magnitude of between-study heterogeneity. Given the anticipated clinical heterogeneity within the hemodialysis population, we will apply a random-effects model for all meta-analyses to provide more conservative estimates, regardless of the level of statistical heterogeneity (I^2^) [33]. We will explore sources of heterogeneity through subgroup analyses or sensitivity analyses, focusing on factors such as age, sex, sample size, dialysis modality, diagnostic tools, dialysis vintage, and comorbidities. To specifically address heterogeneity potentially arising from different measurement instruments, we will conduct subgroup analyses based on the diagnostic tool used (e.g., PSQI, AIS, ISI, or clinical diagnostic criteria) to determine if the choice of instrument acts as a source of heterogeneity or influences the strength of the observed associations. We will examine whether the strength of associated factors varies depending on the outcome measurement method employed. If significant heterogeneity (I^2^ > 75%) exists and cannot be explained by subgroup analyses, we will prioritize descriptive analysis over quantitative pooling, focusing on the consistency of the direction of effects, and we will discuss these limitations. In the narrative synthesis, we will strictly adhere to the Synthesis Without Meta-analysis (SWiM) reporting guidelines to conduct a transparent narrative synthesis [34].

### Quality of evidence and publication bias

Two reviewers (SA and XL) will evaluate the certainty of evidence using the GRADE approach [35]. Given that this review focuses on identifying associated factors, we will adapt the GRADE framework for prognostic factor research to appropriately assess the certainty of evidence [36]. Evidence will be downgraded for limitations in risk of bias, inconsistency, indirectness, imprecision, and publication bias. Upgrading may occur for large effect sizes or dose-response gradients. If ≥ 10 studies are included, publication bias will be assessed using funnel plot visual inspection and Egger’s test (*P < 0.10* indicates bias). If asymmetry suggests reporting bias, the trim-and-fill method will be used for adjustment.

### Sensitivity analysis

We will perform sensitivity analyses to evaluate the robustness of the pooled results by using the leave-one-out method and by excluding low-quality studies to determine whether the inclusion of such studies significantly alters the findings. If sufficient studies are available (≥10), meta-regression will be conducted using Stata to investigate sources of heterogeneity, with effect size as the dependent variable and study characteristics (e.g., sample size, dialysis duration, quality score) as independent variables.

### Study status and timeline

As of December 2025, preliminary scoping searches have been conducted solely to develop the search strategy and define eligibility criteria. Formal data collection and screening have not yet begun. To ensure methodological rigor, we plan to execute the comprehensive literature search and study selection immediately upon the formal acceptance of this protocol. (1) Completion of participant recruitment: Not applicable; (2) Completion of data collection: We anticipate completing the formal literature search and data extraction within five months of the start date (approximately by July 2026); (3) Publication of expected results: Statistical analysis and manuscript writing are anticipated to be finalized by November 2026.

## Discussion

Although numerous observational studies have explored potential associated factors for insomnia in HD patients, current evidence remains fragmented and inconsistent. For instance, some studies suggest that high-flux dialysis modalities may improve sleep quality by enhancing toxin clearance [28], while others report that dialysis modality has a negligible impact on sleep disorders [37]. Similarly, the association between biochemical markers and sleep quality is controversial, with conflicting findings regarding the significance of the effect [37,38]. These inconsistencies may stem from varying sample sizes, diverse population characteristics, and a lack of adjustment for confounding factors in primary studies. Due to the lack of high-quality evidence synthesis, identifying reliable targets for intervention remains challenging. To our knowledge, this will be the first systematic review and meta-analysis to comprehensively evaluate the associated factors for insomnia in HD patients.

This study possesses several methodological strengths. First, we will employ a broad search strategy across multiple English and Chinese databases, including grey literature, to minimize publication and language biases. Secondly, we will strictly adhere to the PRISMA-P guidelines to ensure methodological rigor. Unlike previous reviews that might exclude studies solely based on quality scores, we will retain studies with varying risks of bias and perform sensitivity analyses to test the robustness of our results. Finally, to evaluate the strength of our findings, we will utilize the GRADE framework adapted for prognostic factor research to grade the certainty of evidence. This approach allows us to clearly distinguish which associated factors are supported by high certainty evidence and which are limited by factors such as excessive heterogeneity or risk of bias.

The findings of this review will have significant clinical implications. By distinguishing and identifying modifiable associated factors (e.g., dialysis modality, dialysis shift, lifestyle), non-modifiable associated factors (e.g., age, dialysis vintage), and accompanying symptoms associated with insomnia (e.g., pruritus, depression, RLS), our results will assist healthcare professionals in developing targeted health promotion plans, ultimately alleviating the burden of insomnia in this vulnerable population. Furthermore, we anticipate significant clinical heterogeneity in population characteristics, insomnia definitions, and exposure measurements among the included studies. We will follow the principle that direction is more important than magnitude. That is, if the meta-analysis reveals high heterogeneity (50% < I^2^ ≤ 75%), we will prioritize evaluating the consistency of the effect direction rather than overemphasizing the precision of the pooled effect size. Specifically, if the direction of association is consistent across studies despite varying magnitudes, we will conclude a probable association exists but will be cautious regarding the precise odds ratio. Conversely, if the direction varies or quantitative pooling is inappropriate, we will conduct a transparent narrative synthesis. Finally, this review aims to integrate existing evidence to generate scientific hypotheses and identify potential intervention targets, providing a theoretical basis for future randomized controlled trials (RCTs). We advocate for future research to prioritize modifiable factors. Elucidating the associations between these factors and insomnia is crucial for developing precision management strategies aimed at improving sleep quality in hemodialysis patients.

We anticipate several limitations. First, due to the observational nature of the included studies, our meta-analysis can only establish associations and cannot determine causality. Second, given that factors associated with insomnia may be influenced by dialysis modality, geographic/cultural factors, and diagnostic tools, we expect significant statistical heterogeneity. We plan to address this through pre-specified subgroup analyses and meta-regression, and we will use narrative description to explain findings when quantitative pooling is inappropriate. Third, although we included major Chinese databases to reduce language bias, the use of translation tools for preliminary screening of other non-English languages may introduce slight inaccuracies, although we will mitigate this through manual verification. Finally, due to a limited number of studies or inconsistent data reporting, certain associated factors may preclude quantitative synthesis; in such cases, we will employ narrative synthesis, which may limit the strength of the pooled estimates.

## Conclusion

Strictly adhering to international guidelines such as PRISMA, NOS, MOOSE, and GRADE, this study aims to ensure reporting standards are met. By comprehensively exploring epidemiological data across different regions and cultures, this study focuses on identifying key modifiable associated factors and potential protective factors. These findings will provide vital guidance for clinical practice, facilitating the development of evidence-based disease prevention and health management strategies to ultimately improve sleep quality and overall prognosis in hemodialysis patients.

## Supporting information

S1 File PRISMA-P checklist. (PDF)

S2 File Search strategy. (DOCX)

S3 File MOOSE (Meta-analyses Of Observational Studies in Epidemiology) Checklist. (PDF)

S4 File The Newcastle Ottawa quality assessment instrument (NOS). (DOC)
