# Supplementary material for: Factors associated with insomnia in hemodialysis patients: A protocol for systematic review and meta-analysis
Source: PLoS One. 2026 Feb 20;21(2):e0343236. doi: 10.1371/journal.pone.0343236 (PMC12922989; doi:10.1371/journal.pone.0343236)
Supplement: S2 File — (DOCX) [file pone.0343236.s002.docx]

**S2 File Search strategy**

| **PubMed** | |
| --- | --- |
| # | Query |
| #1 | “Renal Dialysis”[Mesh] |
| #2 | “Dialyses, Renal”[Title/Abstract] OR “Renal Dialyses”[Title/Abstract] OR “Dialysis, Renal”[Title/Abstract] OR “Hemodialysis”[Title/Abstract] OR “Hemodialyses”[Title/Abstract] OR “Dialysis, Extracorporeal”[Title/Abstract] OR “Dialyses, Extracorporeal”[Title/Abstract] OR “Extracorporeal Dialyses”[Title/Abstract] OR “Extracorporeal Dialysis”[Title/Abstract] |
| #3 | #1 OR #2 |
| #4 | “Sleep Initiation and Maintenance Disorders”[Mesh] |
| #5 | "DIMS"[Title/Abstract] OR "Disorders of Initiating and Maintaining Sleep"[Title/Abstract] OR "Sleeplessness"[Title/Abstract] OR "Insomnia Disorder"[Title/Abstract] OR "Insomnia Disorders"[Title/Abstract] OR "Insomnia"[Title/Abstract] OR "Insomnias"[Title/Abstract] OR "Chronic Insomnia"[Title/Abstract] OR "Early Awakening"[Title/Abstract] OR "Nonorganic Insomnia"[Title/Abstract] OR "Primary Insomnia"[Title/Abstract] OR "Psychophysiological Insomnia"[Title/Abstract] OR "Rebound Insomnia"[Title/Abstract] OR "Secondary Insomnia"[Title/Abstract] OR "Sleep Initiation Dysfunction"[Title/Abstract] OR "Sleep Initiation Dysfunctions"[Title/Abstract] OR "Transient Insomnia"[Title/Abstract] |
| #6 | #4 OR #5 |
| #7 | “Risk Factors”[Mesh] |
| #8 | (“Risk Factors”[Title/Abstract] OR “Factor, Risk”[Title/Abstract] OR “Risk Factor”[Title/Abstract] OR “Population at Risk”[Title/Abstract] OR “Populations at Risk”[Title/Abstract] OR “Risk Scores”[Title/Abstract] OR “Risk Score”[Title/Abstract] OR “Score, Risk”[Title/Abstract] OR “Risk Factor Scores”[Title/Abstract] OR “Risk Factor Score”[Title/Abstract] OR “Score, Risk Factor”[Title/Abstract] OR “Health Correlates”[Title/Abstract] OR “Correlates, Health”[Title/Abstract] OR “Social Risk Factors”[Title/Abstract] OR “Factors, Social Risk”[Title/Abstract] OR “Risk Factor, Social”[Title/Abstract] OR “Risk Factors, Social”[Title/Abstract] OR “Social Risk Factor”[Title/Abstract] OR "Associated Factors"[Title/Abstract] OR "Association"[Title/Abstract] OR "Associations"[Title/Abstract] OR "Correlates"[Title/Abstract] OR "Predictors"[Title/Abstract] OR "Predictive Factors"[Title/Abstract] OR "Determinants"[Title/Abstract] OR "Precipitating Factors"[Title/Abstract]) |
| #9 | #7 OR #8 |
| #10 | #3 AND #6 AND #9 |
| **Web of Science** | |
| #1 | TS= ("Renal Dialysis" OR "Renal Dialyses" OR "Hemodialysis" OR "Hemodialyses" OR "Extracorporeal Dialysis" OR "Extracorporeal Dialyses") |
| #2 | TS= ("Sleep Initiation and Maintenance Disorders" OR "DIMS" OR "Disorders of Initiating and Maintaining Sleep" OR "Sleeplessness" OR "Insomnia*" OR "Early Awakening" OR "Sleep Initiation Dysfunction*" OR "Nonorganic Insomnia" OR "Psychophysiological Insomnia") |
| #3 | TS= ("Risk Factor*" OR "Population* at Risk" OR "Risk Score*" OR "Health Correlate*" OR "Social Risk Factor*" OR "Associated Factor*" OR "Association*" OR "Correlate*" OR "Predictor*" OR "Determinant*") |
| #4 | #1 AND #2 AND #3 |
| **CINAHL** | |
| #1 | (MH "Hemodialysis") OR (MH "Renal Replacement Therapy") OR TI ("Renal Dialysis" OR "Renal Dialyses" OR "Hemodialysis" OR "Hemodialyses" OR "Extracorporeal Dialysis" OR "Extracorporeal Dialyses") OR AB ("Renal Dialysis" OR "Renal Dialyses" OR "Hemodialysis" OR "Hemodialyses" OR "Extracorporeal Dialysis" OR "Extracorporeal Dialyses") |
| #2 | (MH "Insomnia") OR TI ("DIMS" OR "Disorders of Initiating and Maintaining Sleep" OR "Sleeplessness" OR "Insomnia" OR "Insomnias" OR "Chronic Insomnia" OR "Early Awakening" OR "Nonorganic Insomnia" OR "Primary Insomnia" OR "Psychophysiological Insomnia" OR "Rebound Insomnia" OR "Secondary Insomnia" OR "Sleep Initiation Dysfunction" OR "Transient Insomnia") OR AB ("DIMS" OR "Disorders of Initiating and Maintaining Sleep" OR "Sleeplessness" OR "Insomnia" OR "Insomnias" OR "Chronic Insomnia" OR "Early Awakening" OR "Nonorganic Insomnia" OR "Primary Insomnia" OR "Psychophysiological Insomnia" OR "Rebound Insomnia" OR "Secondary Insomnia" OR "Sleep Initiation Dysfunction" OR "Transient Insomnia") |
| #3 | (MH "Risk Factors") OR TI ("Risk Factor" OR "Risk Factors" OR "Population at Risk" OR "Populations at Risk" OR "Risk Score" OR "Risk Scores" OR "Health Correlates" OR "Social Risk Factors" OR "Associated Factors" OR "Associated Factor" OR "Association" OR "Associations" OR "Correlates" OR "Predictors" OR "Predictive Factors" OR "Determinants" OR "Precipitating Factors") OR AB ("Risk Factor" OR "Risk Factors" OR "Population at Risk" OR "Populations at Risk" OR "Risk Score" OR "Risk Scores" OR "Health Correlates" OR "Social Risk Factors" OR "Associated Factors" OR "Associated Factor" OR "Association" OR "Associations" OR "Correlates" OR "Predictors" OR "Predictive Factors" OR "Determinants" OR "Precipitating Factors") |
| #4 | #1 AND #2 AND #3 |
| **Cochrane Library** | |
| #1 | [mh "Renal Dialysis"] OR ("Renal Dialysis" OR "Renal Dialyses" OR "Dialysis, Renal" OR "Hemodialysis" OR "Hemodialyses" OR "Extracorporeal Dialysis" OR "Extracorporeal Dialyses"):ti,ab,kw |
| #2 | [mh "Sleep Initiation and Maintenance Disorders"] OR ("DIMS" OR "Disorders of Initiating and Maintaining Sleep" OR "Sleeplessness" OR "Insomnia" OR "Insomnias" OR "Chronic Insomnia" OR "Early Awakening" OR "Nonorganic Insomnia" OR "Primary Insomnia" OR "Psychophysiological Insomnia" OR "Rebound Insomnia" OR "Secondary Insomnia" OR "Sleep Initiation Dysfunction" OR "Sleep Initiation Dysfunctions" OR "Transient Insomnia"):ti,ab,kw |
| #3 | [mh "Risk Factors"] OR ("Risk Factors" OR "Factor, Risk" OR "Risk Factor" OR "Population at Risk" OR "Populations at Risk" OR "Risk Scores" OR "Risk Score" OR "Score, Risk" OR "Risk Factor Scores" OR "Risk Factor Score" OR "Score, Risk Factor" OR "Health Correlates" OR "Correlates, Health" OR "Social Risk Factors" OR "Factor, Social Risk" OR "Factors, Social Risk" OR "Risk Factor, Social" OR "Risk Factors, Social" OR "Social Risk Factor" OR (Associated NEXT Factor) OR Association OR Correlate* OR Predictor* OR (Predictive NEXT Factor*) OR Determinant* OR (Precipitating NEXT Factor*)**):ti,ab,kw |
| #4 | #1 AND #2 AND #3 |
| **EMBASE** | |
| #1 | 'insomnia'/exp OR ('dims' OR 'disorders of initiating and maintaining sleep' OR 'sleeplessness' OR 'insomnia' OR 'insomnias' OR 'chronic insomnia' OR 'early awakening' OR 'nonorganic insomnia' OR 'primary insomnia' OR 'psychophysiological insomnia' OR 'rebound insomnia' OR 'secondary insomnia' OR 'sleep initiation dysfunction' OR 'sleep initiation dysfunctions' OR 'transient insomnia'):ti,ab,kw |
| #2 | 'hemodialysis'/exp OR ('renal dialysis' OR 'renal dialyses' OR 'dialysis, renal' OR 'hemodialysis' OR 'hemodialyses' OR 'extracorporeal dialysis' OR 'extracorporeal dialyses'):ti,ab,kw |
| #3 | 'risk factor'/exp OR ('risk factors' OR 'factor, risk' OR 'risk factor' OR 'population at risk' OR 'populations at risk' OR 'risk scores' OR 'risk score' OR 'score, risk' OR 'risk factor scores' OR 'risk factor score' OR 'score, risk factor' OR 'health correlates' OR 'correlates, health' OR 'social risk factors' OR 'factor, social risk' OR 'factors, social risk' OR 'risk factor, social' OR 'risk factors, social' OR 'social risk factor' OR 'associated factor' OR 'association' OR 'correlate*' OR 'predictor*' OR 'predictive factor*' OR 'determinant*' OR 'precipitating factor*'):ti,ab,kw |
| #4 | #1 AND #2 AND #3 |
| **CNKI** | |
| #1 | SU=(肾透析 + 体外透析 + 血液透析 + 连续性血液净化 + 血透 + 维持性血液透析 + 维持性血透 + 连续血液净化 + 持续血液净化) |
| #2 | TKA=(肾透析 + 体外透析 + 血液透析 + 连续性血液净化 + 血透 + 维持性血液透析 + 维持性血透 + 连续血液净化 + 持续血液净化) |
| #3 | KY=(肾透析 + 体外透析 + 血液透析 + 连续性血液净化 + 血透 + 维持性血液透析 + 维持性血透 + 连续血液净化 + 持续血液净化) |
| #4 | #1 OR #2 OR #3 |
| #5 | SU=(入睡和睡眠障碍 + 反跳性失眠症 + 继发性失眠 + 睡眠起始功能障碍 + 失眠 + 失眠症 + 慢性失眠 + 早醒 + 非器质性失眠症 + 原发性失眠 + 入睡和睡眠失调 + DIMS + 心理生理性失眠 + 暂时性失眠 + 不寐) |
| #6 | TKA=(入睡和睡眠障碍 + 反跳性失眠症 + 继发性失眠 + 睡眠起始功能障碍 + 失眠 + 失眠症 + 慢性失眠 + 早醒 + 非器质性失眠症 + 原发性失眠 + 入睡和睡眠失调 + DIMS + 心理生理性失眠 + 暂时性失眠 + 不寐) |
| #7 | KY=(入睡和睡眠障碍 + 反跳性失眠症 + 继发性失眠 + 睡眠起始功能障碍 + 失眠 + 失眠症 + 慢性失眠 + 早醒 + 非器质性失眠症 + 原发性失眠 + 入睡和睡眠失调 + DIMS + 心理生理性失眠 + 暂时性失眠 + 不寐) |
| #8 | #5 OR #6 OR #7 |
| #9 | SU=(危险因素 + 影响因素 + 预测因素 + 相关因素 + 相关性 + 关联因素) |
| #10 | TKA=(危险因素 + 影响因素 + 预测因素 + 相关因素 + 相关性 + 关联因素) |
| #11 | KY=(危险因素 + 影响因素 + 预测因素 + 相关因素 + 相关性 + 关联因素) |
| #12 | #9 OR #10 OR #11 |
| #13 | #4 AND #8 AND #12 |
| **WanFang** | |
| #1 | 主题:(肾透析 OR 体外透析 OR 血液透析 OR 连续性血液净化 OR 血透 OR 维持性血液透析 OR 维持性血透 OR 连续血液净化 OR 持续血液净化) |
| #2 | 题名或关键词:(肾透析 OR 体外透析 OR 血液透析 OR 连续性血液净化 OR 血透 OR 维持性血液透析 OR 维持性血透 OR 连续血液净化 OR 持续血液净化) |
| #3 | #1 OR #2 |
| #4 | 主题:(入睡和睡眠障碍 OR 反跳性失眠症 OR 继发性失眠 OR 睡眠起始功能障碍 OR 失眠 OR 失眠症 OR 慢性失眠 OR 早醒 OR 非器质性失眠症 OR 原发性失眠 OR 入睡和睡眠失调 OR DIMS OR 心理生理性失眠 OR 暂时性失眠 OR 不寐) |
| #5 | 题名或关键词:(主题:(入睡和睡眠障碍 OR 反跳性失眠症 OR 继发性失眠 OR 睡眠起始功能障碍 OR 失眠 OR 失眠症 OR 慢性失眠 OR 早醒 OR 非器质性失眠症 OR 原发性失眠 OR 入睡和睡眠失调 OR DIMS OR 心理生理性失眠 OR 暂时性失眠 OR 不寐)) |
| #6 | #4 OR #5 |
| #7 | 主题:(危险因素 OR 影响因素 OR 预测因素 OR 相关因素 OR 相关性 OR 关联因素) |
| #8 | 题名或关键词:(危险因素 OR 影响因素 OR 预测因素 OR 相关因素 OR 相关性 OR 关联因素) |
| #9 | #7 OR #8 |
| #10 | #3 AND #6 AND #9 |
| **SinoMed** | |
| #1 | ( "肾透析"[加权:扩展] OR "体外透析"[加权:扩展] OR "血液透析"[加权:扩展] OR "连续性血液净化"[加权:扩展] OR "血透"[加权:扩展] OR "维持性血液透析"[加权:扩展] OR "维持性血透"[加权:扩展] OR "连续血液净化"[加权:扩展] OR "持续血液净化"[加权:扩展]) |
| #2 | "("[关键词:智能] AND ""肾透析"[加权:扩展]"[关键词:智能] AND "OR"[关键词:智能] AND ""体外透析"[加权:扩展]"[关键词:智能] AND "OR"[关键词:智能] AND ""血液透析"[加权:扩展]"[关键词:智能] AND "OR"[关键词:智能] AND ""连续性血液净化"[加权:扩展]"[关键词:智能] AND "OR"[关键词:智能] AND ""血透"[加权:扩展]"[关键词:智能] AND "OR"[关键词:智能] AND ""维持性血液透析"[加权:扩展]"[关键词:智能] AND "OR"[关键词:智能] AND ""维持性血透"[加权:扩展]"[关键词:智能] AND "OR"[关键词:智能] AND ""连续血液净化"[加权:扩展]"[关键词:智能] AND "OR"[关键词:智能] AND ""持续血液净化"[加权:扩展])"[关键词:智能] |
| #3 | #1 OR #2 |
| #4 | ( "入睡和睡眠障碍"[加权:扩展] OR "反跳性失眠症"[加权:扩展] OR "继发性失眠"[加权:扩展] OR "睡眠起始功能障碍"[加权:扩展] OR "失眠"[加权:扩展] OR "失眠症"[加权:扩展] OR "慢性失眠"[加权:扩展] OR "早醒"[加权:扩展] OR "非器质性失眠症"[加权:扩展] OR "原发性失眠"[加权:扩展] OR "入睡和睡眠失调"[加权:扩展] OR "DIMS"[加权:扩展] OR "心理生理性失眠"[加权:扩展] OR "暂时性失眠"[加权:扩展] OR "不寐"[加权:扩展]) |
| #5 | ( "入睡和睡眠障碍"[关键词:智能] OR "反跳性失眠症"[关键词:智能] OR "继发性失眠"[关键词:智能] OR "睡眠起始功能障碍"[关键词:智能] OR "失眠"[关键词:智能] OR "失眠症"[关键词:智能] OR "慢性失眠"[关键词:智能] OR "早醒"[关键词:智能] OR "非器质性失眠症"[关键词:智能] OR "原发性失眠"[关键词:智能] OR "入睡和睡眠失调"[关键词:智能] OR "DIMS"[关键词:智能] OR "心理生理性失眠"[关键词:智能] OR "暂时性失眠"[关键词:智能] OR "不寐"[关键词:智能]) |
| #6 | #4 OR #5 |
| #7 | ( "危险因素"[加权:扩展] OR "影响因素"[加权:扩展] OR "预测因素"[加权:扩展] OR "相关因素"[加权:扩展] OR "相关性"[加权:扩展] OR "关联因素"[加权:扩展]) |
| #8 | ( "危险因素"[关键词:智能] OR "影响因素"[关键词:智能] OR "预测因素"[关键词:智能] OR "相关因素"[加权:扩展] OR "相关性"[加权:扩展] OR "关联因素"[加权:扩展]) |
| #9 | #7 OR #8 |
| #10 | #3 AND #6 AND #9 |
|  | Google Scholar（Taking English search terms as an example） |
| #1 | ("Hemodialysis" OR "Renal Dialysis") AND ("Insomnia" OR "Sleep Initiation and Maintenance Disorders" OR "Sleep Quality") AND ("Risk Factors" OR "Predictors" OR "Associated Factors" OR "Correlates") |
